# Time Estimation Predicts Mathematical Intelligence

**DOI:** 10.1371/journal.pone.0028621

**Published:** 2011-12-07

**Authors:** Peter Kramer, Paola Bressan, Massimo Grassi

**Affiliations:** Dipartimento di Psicologia Generale, University of Padova, Padova, Italy; Katholieke Universiteit Leuven, Belgium

## Abstract

**Background:**

Performing mental subtractions affects time (duration) estimates, and making time estimates disrupts mental subtractions. This interaction has been attributed to the concurrent involvement of time estimation and arithmetic with general intelligence and working memory. Given the extant evidence of a relationship between time and number, here we test the stronger hypothesis that time estimation correlates specifically with mathematical intelligence, and not with general intelligence or working-memory capacity.

**Methodology/Principal Findings:**

Participants performed a (prospective) time estimation experiment, completed several subtests of the WAIS intelligence test, and self-rated their mathematical skill. For five different durations, we found that time estimation correlated with both arithmetic ability and self-rated mathematical skill. Controlling for non-mathematical intelligence (including working memory capacity) did not change the results. Conversely, correlations between time estimation and non-mathematical intelligence either were nonsignificant, or disappeared after controlling for mathematical intelligence.

**Conclusions/Significance:**

We conclude that time estimation specifically predicts mathematical intelligence. On the basis of the relevant literature, we furthermore conclude that the relationship between time estimation and mathematical intelligence is likely due to a common reliance on spatial ability.

## Introduction

Circadian rhythms regulate sleep, body temperature, and the functioning of various organs [1], demonstrating the importance of implicit time estimation to biological systems. Meanwhile, explicit (e.g., verbal) time estimation can reveal psychopathology [2], [3] and expose memory capacity limits [4], [5] which are in turn related to general intelligence [6], [7]. Here, we investigate prospective time estimation (i.e., with the task known in advance) of 100- to 3000-millisecond durations under minimal working-memory load. With subtests of the Wechsler Adult Intelligence Scale (WAIS-R), we measure both mathematical and non-mathematical intelligence, as well as working-memory capacity. We show that time estimation under low working-memory load correlates specifically with mathematical, and not with general (non-mathematical) intelligence.

Our research is motivated by direct and indirect evidence of a tight link (a) between temporal and numerical processing and (b) between even the simplest numerical processing and mathematical intelligence. Several authors have suggested that the processing of spatial, numerical, and temporal information involve either tightly intertwined magnitude representations or a single, common one [8]–[14].

More specifically, it has been suggested that numbers are represented along a left-to-right mental number line [15] and durations along a left-to-right mental time line [16]. In numerical processing, reaction time in number comparison decreases with the numerical distance between numbers [17], suggesting indeed a spatial representation. In temporal processing, consistently, auditory duration estimates increase with concurrently perceived visual length (whereas loudness estimates do not [18], hence excluding a response-competition explanation).

In numerical processing, the Spatial Numerical Association of Response Codes (SNARC) effect provides additional evidence for a spatial representation of numerical magnitude. In number-parity judgment (odd vs. even), for example, left-side responses are faster to small than to large numbers, whereas right-side responses are faster to large than to small numbers [19]. Although, in principle, the SNARC effect can be explained without assuming a spatial-numerical representation [20], related effects cannot [21], [22]. In temporal processing, the Spatial TEmporal Association of Response Codes (STEARC) effect provides similar evidence: when judging whether a final duration is shorter or longer than a repeated standard, short durations induce faster left- than right-side responses, whereas long ones induce faster right- than left-side responses ([16]; for a related effect, see [23]). Furthermore, prior adaptation to wearing prisms decreases visual duration estimates for leftward prisms and increases them for rightward prisms ([24]; for related effects, see [25]–[27]).

Neuropsychological evidence on hemispatial neglect lends further support to the conjecture that numbers and durations are both spatially represented. Hemispatial neglect consists in a deficit in attending to the left hemispace following right inferior parietal lesions [28]. Whereas the hallmark of the disorder concerns deficits in spatial attention, concurrent deficits have been observed in the processing of size, number, and time [14], [29]–[32]. These deficits, moreover, can be induced in healthy subjects via transcranial magnetic stimulation of the same area [14].

When asked to bisect a line, neglect patients typically display a rightward bias. A striking example of the spatial nature of numerical representations is that neglect patients, while reporting which number falls exactly in between two others, also show a rightward bias on their mental number line. That is, when asked to report which number falls exactly in between 2 and 6, they typically report 5 instead of 4 ([33]; for related studies in normals, see [34]–[36]). In temporal processing, consistent with a spatial representation of time, hemispatial neglect has been found to lead to overestimation of durations in the neglected space and underestimation elsewhere [37].

Direct behavioral evidence of interactions between numerical and temporal processing exists too. Some of these interactions may be due to response competition [18], but some cannot. Brown [4], for example, found that concurrent elementary arithmetic decreased time estimates, and vice versa, and that pursuit rotor tracking and visual search affected time estimation too, but not vice versa. The arithmetic only involved basic subtractions and Brown did not connect his findings to mathematical intelligence. Instead, he argued that arithmetic competed more strongly than pursuit and visual search for both working memory and general-purpose processing resources. Fink and Neubauer [7] found that time estimates during simple additions and subtractions improved with intelligence. These authors too, however, attributed the effect of basic arithmetic to working memory capacity, general-purpose processing, and general rather than mathematical intelligence.

A skill that requires little if any working memory capacity, or general-purpose processing resources, is numerosity (discrete quantity) estimation. Since it does not involve symbolic processing, it is a very basic skill. Yet, it has been shown to specifically predict mathematical ability, and not other kinds of competence [38]; it is also associated to the mathematical disability of dyscalculia [39]. With the literature suggesting a tight link between temporal and numerical processing, our hypothesis presents itself quite naturally: time (duration) estimation should correlate specifically with mathematical, rather than non-mathematical, intelligence, and should not necessarily be affected by working memory capacity.

## Materials and Methods

### Ethics statement

The experimental procedures were approved by the Institutional Review Board at the University of Padova, and were in accordance with the Declaration of Helsinki (Sixth Revision, 2008). All participants gave their informed written consent to participate in the study.

### Participants

The participants were 202 naïve students (101 women and 101 men, mean age 22 years, range 18–52 years), who were recruited and tested individually. All participants reported normal hearing.

### Apparatus

The experiment was implemented in Matlab (Mathworks ©). The software was running on a Pentium IV computer connected to a NEC Multisync FP950 monitor and an M-AUDIO Fast Track Pro sound card. The output of the sound card was delivered to the subject via Sennheiser HD 560 headphones at 65 dBA pressure level measured at the subject's ear. Sounds presented during the experiment had 16-bit resolution and a sample rate of 44.1 kHz.

### Stimuli, materials, and procedure

Participants performed an auditory prospective time-estimation task (which depends less on memory than a retrospective one [40]), followed by four subtests of the WAIS-R (the Wechsler Adult Intelligence Scale Revised). Finally, subjects rated their mathematical skill subjectively on an 11-point Likert scale that ranged from 0 (very poor) to 10 (excellent), a range identical to that customarily used in Italian school grading.

We first made sure that participants knew that one millisecond is a thousandth of a second; next, we presented them a series of tones. The tones were amplitude-steady complex ones, gated on and off with 10-ms raised cosine ramps (to avoid onset and offset clicks), including the first four harmonics of a 250-Hz fundamental. After each tone, participants typed their estimate of its duration in milliseconds. The tone durations were 100, 200, 500, 1000, and 3000 ms (spanning the range of so called *interval timing*; [8]), replicated six times each and presented in random order. There were no secondary tasks and working memory load was thus minimal.

Intelligence was measured with the Italian version of the arithmetic, digit span forward, digit span backward, and similarities subtests of the WAIS-R. The arithmetic subtest involves solving arithmetic problems from easy (e.g., “What is the total of 4 plus 5 apples?”) to relatively hard (e.g., “If 8 machines can finish a job in 6 days, how many machines are needed to finish it in half a day?”). The digit span forward subtest requires the repetition of 3 to 9 digits. The digit span backward subtest requires the repetition of 2 to 8 digits in reverse order. The similarities subtest requires solving non-mathematical problems from easy (“In what way are an orange and a banana alike?”) to relatively hard (“In what way are praise and punishment alike?”). The arithmetic subtest is expected to measure mathematical intelligence, the digit span subtests are expected to measure working-memory capacity, and the similarities subtest is sensitive to general or non-mathematical intelligence.

## Results

For each subject, we averaged across the six time estimates for each of the five tone durations. For each of the resulting average time estimates, we then calculated the absolute standardized time estimation error (henceforth *time estimation error*): | ψ – φ | / φ, with ψ denoting psychological (estimated) duration and φ physical duration. Table 1 shows the descriptive statistics of the WAIS-R subscales and self-rated mathematical skill.

**Table 1:** Descriptive statistics of the WAIS-R subscales and self-rated mathematical skill.

Intelligence tests	Range	Mean	Median	Std. dev.
Arithmetic	0–19	10.94	11	3.37
Self-rated math skill	0–10	5.47	6	1.89
Digit span forward	0–14	7.88	8	1.83
Digit span backward	0–14	7.18	7	1.82
Similarities	0–28	19.61	20	3.09

*Note.* “Std. dev.” stands for “standard deviation”.

### Correlations

Time estimations for the five durations were highly inter-correlated. The correlations ranged from .30 to .83, with an average of .59. Hence, we only considered simple (Pearson) correlations rather than multiple correlations (Table 2). Time estimation errors correlated negatively with arithmetic scores and, except for the 3000-ms duration, also with self-rated mathematical skill and digit span forward. No other correlations reached even marginal significance. Excluding data points that were three standard deviations away from the mean did not change the pattern of results, except that, for the 100-ms duration, the correlation between time estimation and digit span forward no longer reached (marginal) significance. (The subjects' self-rated mathematical intelligence scores ranged from 0 to 9; the highest score of 10 was never chosen.)

**Table 2:** Pearson correlations between intelligence and time-estimation error for five different tone durations.

	Tone durations in milliseconds				
Intelligence tests	100	200	500	1000	3000
Arithmetic	−.28 (.000)	−.26 (.000)	−.25 (.000)	−.29 (.000)	−.22 (.002)
Self-rated math skill	−.31 (.000)	−.31 (.000)	−.30 (.000)	−.19 (.008)	−.10 (.168)
Digit span forward	−.14 (.040)	−.14 (.045)	−.18 (.011)	−.17 (.013)	−.11 (.125)
Digit span backward	−.09 (.225)	−.07 (.343)	−.07 (.325)	−.11 (.123)	−.12 (.087)
Similarities	−.01 (.926)	−.01 (.916)	−.00 (.973)	−.01 (.941)	−.05 (.478)

*Note.* The Pearson correlations are presented with their p-values between brackets. *N* = 202 for all correlations, except those involving digit span backward, for which *N* = 201 (one subject failed to fill out this subtest). Spearman correlations were similar.

### Partial correlations

When all measures of non-mathematical intelligence (digit span forward, digit span backward, and similarities) were partialled out, all the significant negative correlations between time estimation error and either arithmetic or self-rated mathematical skill remained significant (Table 3). The correlations also remained significant after partialling out age and sex. Conversely, when the two measures of mathematical intelligence (arithmetic and self-rated mathematical skill) were partialled out, none of the correlations between time estimation and non-mathematical intelligence (digit span forward, digit span backward, similarities) reached even marginal significance (Table 3).

**Table 3:** Partial correlations between intelligence and time-estimation error for five different tone durations.

	Tone durations in milliseconds				
Intelligence tests	100	200	500	1000	3000
Arithmetic	−.26 (.000)	−.24 (.001)	−.23 (.001)	−.27 (.000)	−.18 (.010)
Self-rated math skill	−.29 (.000)	−.28 (.000)	−.27 (.000)	−.15 (.031)	−.06 (.380)
Digit span forward	−.06 (.392)	−.06 (.367)	−.10 (.142)	−.10 (.171)	−.05 (.488)
Digit span backward	.04 (.602)	.05 (.490)	.04 (.536)	.01 (.924)	−.04 (.561)
Similarities	.08 (.289)	.07 (.338)	.07 (.307)	.08 (.285)	.01 (.933)

*Note.* For arithmetic and self-rated mathematical skill, all measures of non-mathematical intelligence (digit span forward, digit span backward, and similarities) were partialled out (*df* = 196). For the non-mathematical intelligence measures, both arithmetic and self-rated mathematical skill were partialled out (*df* = 197).

**Figure 1:**
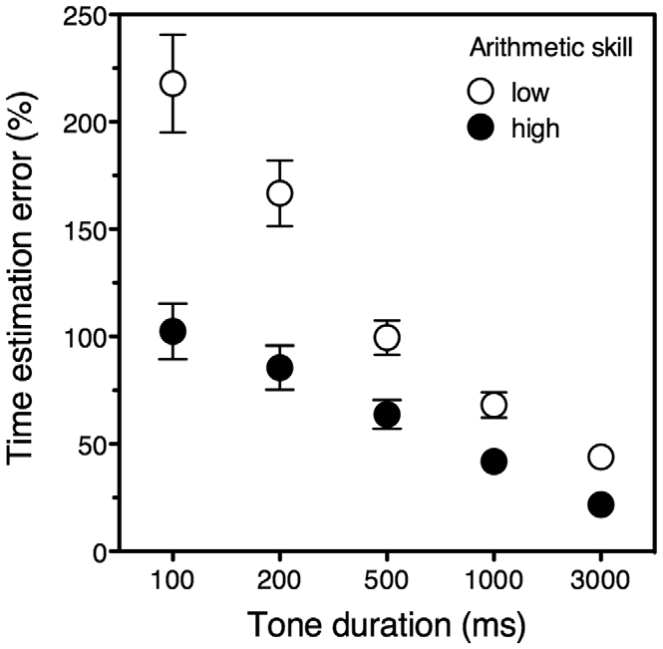
Time estimation error and arithmetic proficiency. Percent absolute standardized time estimation error, at five tone durations, for participants whose arithmetic score fell either in the lowest (open symbols) or highest (closed symbols) tertile. Error bars represent ±1 standard error of the mean.

Figure 1 shows the essence of our findings: participants with higher arithmetic scores were consistently better at time estimation than participants with lower arithmetic scores. Note that both the error magnitude and the difference between top and bottom arithmetic scorers decreased with tone duration. Likely, the larger percent errors at smaller physical durations are due to a constant, duration-independent sensory error [41].

## Discussion

Our results show that time estimation predicts mathematical intelligence (measured either objectively, via the WAIS-R arithmetic, or subjectively, via self-rated mathematical skill), whereas it is unrelated to two other forms of intelligence—working-memory capacity (WAIS-R digit span) and non-mathematical reasoning (WAIS-R similarities). After we partialled out non-mathematical intelligence, all correlations between time estimation and objectively- or subjectively-measured mathematical intelligence remained significant. In contrast, none of the correlations between time estimation and non-mathematical intelligence remained significant after we partialled out mathematical intelligence.

Brown [4] and Fink and Neubauer [7] found that, in dual tasks, time estimation and concurrent basic arithmetic interfere with each other. Rather than attributing this result to interacting temporal and numerical processing, these authors argued that it was due to the limits of general-purpose working memory capacity and general intelligence. In our study we avoided dual tasks, and working memory load during time estimation was low and unlikely to play any role. We found no relation between time estimation and either working memory capacity (as measured by the digit span forward and digit span backward subtests) or non-mathematical intelligence (as measured by both the digit span subtests and the similarities subtest). Instead, we found that the time estimation skill increased specifically with arithmetic intelligence.

Grondin [5] found that the estimation of the interval between pairs of sensory markers (tone bursts or spots of light), was better if, throughout an experimental block, the intervals varied around the same base duration than if they varied around two different base durations. Grondin argued that, for each base duration, subjects maintain a separate representation of interval distribution in memory. Varying base duration would thus amount to increasing memory load. Our results suggests, however, that if only one duration representation needs to be kept in mind, then time estimation depends only on mathematical intelligence, and not on working memory. Thus, time estimation may, but need not necessarily, be affected by working memory capacity.

Electrophysiological and neuroimaging results reveal that the cortical substrates of time and numerical processing show considerable overlap, involving the prefrontal and posterior-parietal cortexes and the intraparietal sulcus (for reviews, see [8], [9], [13], [14]). The posterior parietal cortex of primates, for example, has been found to be activated during explicit time estimation, but also during a numerical task in which a sequence of movements was to be repeated a particular number of times [8]. The angular gyrus within the parietal cortex has been implicated in the innate disability of *dyscalculia* and the acquired disability of *acalculia*, both involving exceptionally poor numerical and mathematical ability [42]. The intraparietal sulcus has been implicated not only in explicit time estimation and numerical processing [8], but also in dyscalculia [39], [42] and acalculia [42]. Moreover, poor mathematical skill has been associated with deficits in implicit temporal processing. In particular, primary-school children who are poor in mathematics have been found to be worse than age-matched controls in global motion perception, despite being normal in dynamic global-form perception [43].

It is unlikely that one's mathematical ability is related to some internal clock, but mathematical ability does rely on numerical processing [39], [42]. Whereas the processing of sequentially presented numerosities could involve an internal clock [10], the processing of simultaneously presented ones most probably does not. Numerical and temporal processing, however, both rely on spatial representations (see introduction). The relationship between time estimation and mathematical intelligence might thus be due to a common reliance on spatial ability. Indeed, spatial ability has repeatedly been found to predict mathematical ability, including basic arithmetical skill, and has been shown to play a role in both dyscalculia and acalculia [42].

On the basis of our current results, we conclude that time estimation predicts mathematical intelligence. Taking the literature into account, we furthermore conclude that the relationship between the two is likely to be due to a common reliance on spatial ability.
